# Col1A-2 Mutation in Osteogenesis Imperfecta Mice Contributes to Long Bone Fragility by Modifying Cell-Matrix Organization

**DOI:** 10.3390/ijms242317010

**Published:** 2023-11-30

**Authors:** Grégoire André, Antoine Chretien, Antoine Demoulin, Mélanie Beersaerts, Pierre-Louis Docquier, Catherine Behets

**Affiliations:** 1 Pole of Morphology, Institute of Experimental and Clinical Research, Université Catholique de Louvain, 1200 Brussels, Belgium; gregoire.andre@uclouvain.be (G.A.); antoine.chretien@uclouvain.be (A.C.); antoine.demoulin@student.uclouvain.be (A.D.); melanie.beersaerts@uclouvain.be (M.B.); 2 Neuromusculoskeletal Lab, Institute of Experimental and Clinical Research, Université Catholique de Louvain, 1200 Brussels, Belgium; pierre-louis.docquier@uclouvain.be

**Keywords:** osteogenesis imperfecta, bone, OIM, collagen, osteocyte lacunae

## Abstract

Osteogenesis imperfecta (OI) is a rare congenital bone dysplasia generally caused by a mutation of one of the type I collagen genes and characterized by low bone mass, numerous fractures, and bone deformities. The collagen organization and osteocyte lacuna arrangement were investigated in the long bones of 17-week-old wildtype (WT, n = 17) and osteogenesis imperfecta mice (OIM, n = 16) that is a validated model of severe human OI in order to assess their possible role in bone fragility. Fractures were counted after in vivo scanning at weeks 5, 11, and 17. Humerus, femur, and tibia diaphyses from both groups were analyzed ex vivo with pQCT, polarized and ordinary light histology, and Nano-CT. The fractures observed in the OIM were more numerous in the humerus and femur than in the tibia, whereas the quantitative bone parameters were altered in different ways among these bones. Collagen fiber organization appeared disrupted, with a lower birefringence in OIM than WT bones, whereas the osteocyte lacunae were more numerous, more spherical, and not aligned in a lamellar pattern. These modifications, which are typical of immature and less mechanically competent bone, attest to the reciprocal alteration of collagen matrix and osteocyte lacuna organization in the OIM, thereby contributing to bone fragility.

## 1. Introduction

Osteogenesis imperfecta (OI) is a rare genetic disease of connective tissues, whose prevalence is 1/10,000 to 1/20,000 births. In most cases, because of an abnormal production of type I collagen, patients present with short stature, fractures, bone deformities, scoliosis, pain, and disability. Extra-skeletal manifestations can also be observed, such as blue sclera, dentinogenesis imperfecta, hearing loss, etc. [1]. Classification based on genetic criteria distinguishes many types of OI [2]. However, the original Sillence’s classification, based on clinical aspects, remains useful. Type I is a light form with fractures and, sometimes, dentinogenesis imperfecta. Type II is associated with perinatal death. Type III is the most severe form compatible with life; patients present numerous bone deformities and fractures, as well as other symptoms. Type IV is the intermediary between Type I and III. In this intermediate category, patients exhibit recurring fractures, osteoporosis, and bone deformities [3].

Type I collagen, the main protein of bone matrix, is a heterotrimer composed of two alpha-1 chains and one alpha-2. A mutation of one of their respective *COL1A1* and *COL1A2* genes is responsible for about 80% of OI cases [4]. Such mutations lead to disturbances in collagen quality and/or quantity. The remaining 20% of cases are caused by posttranscriptional abnormalities or mutations of non-collagenous proteins [5].

The bone matrix comprises two primary components: the organic phase, predominantly composed of 90% type I collagen, and the mineral phase, consisting of hydroxyapatite crystals. The three-dimensional structure of mature bone tissue depends on the lamellar organization of the collagen matrix, which is essential for the proper arrangement of hydroxyapatite crystals and overall bone matrix quality [6].

The bone matrix is apposited by osteoblasts, differentiated from mesenchymal stem cells, which become osteocytes in the bone matrix after passing successive stages, namely osteoblast, osteoid osteocyte, mineralizing osteocyte, and finally mature osteocyte. In the mineralized matrix, osteocytes are embedded inside individual lacunae and are interconnected with each other via dendrites into canaliculi [7], leading to the lacuno–canalicular network (LCN) distributed throughout bone tissue [8]. External mechanical loading has an impact on cellular shape [9], and the signals that they receive depend on LCN geometry and morphology. Osteocytes play a major role in mechanosensation and mechanotransduction, which are essential for bone remodeling. Indeed, they secrete sclerostin, a potent inhibitor of the Wnt/Beta-catenin pathway, and, thereby, can influence bone formation. They are involved in phosphate metabolism, mineralization, matrix degradation [10,11], and osteoclastogenesis through RANKL secretion. They secrete osteoprotegerin, which competes with RANKL for binding to the RANK-receptor, thereby decreasing osteoclast activity; this interaction is a target for osteoporosis treatment [12,13]. They also produce nitric oxide (NO) and prostaglandin E_2_, which regulate osteoclast and osteoblast activity [13].

Osteocytes are oriented with a specific polarity, depending on the direction of collagen apposition, mineral formation, and loads [14]. They contribute to bone quality and mechanical competence through their organization inside the matrix, as shown in the femur of human infants and children [15]. Moreover, their viability is correlated to bone quality [16]. Finally, spherical osteocytes are known to produce more NO under mechanical loading than flat osteocytes [14].

The aim of the present study is to characterize the organization of extracellular matrix and osteocyte lacunae (OL) in long bones of 17-week-old wildtype (WT) and osteogenesis imperfecta mice (OIM) known to display type III osteogenesis imperfecta features [17]. We hypothesized that, in addition to low bone mass parameters, the organization of bone matrix and OL contributes to bone fragility and fractures in the osteogenesis imperfecta mice. Fractures were counted with 3D in vivo scan NanoSPECT/CT^PLUS^ (Mediso, Budapest, Hungary); thereafter, the humerus, femur, and tibia were analyzed ex vivo with peripheral Quantitative Computed Tomography (pQCT) to assess bone mineral density and quantitative cortical bone parameters, microscopic birefringence to evaluate the collagen organization, as well as histology and Nano-CT to characterize the OL in cortical bone.

## 2. Results

### 2.1. Clinimetry

**Figure 1:**
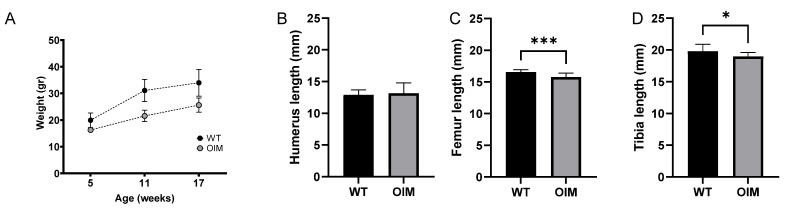
Clinimetric observations. (**A**) Weight (g) of WT and OIM according to age (weeks). The mice were weighed every week, but only the weight at the three times corresponding to the scans is displayed. (**B**–**D**) Length (mm) of the humerus (WT n = 15; OIM n = 8), femur (WT n = 14; OIM n = 10), and tibia (WT n = 15; OIM n = 13) measured at 17 weeks. Results are shown with mean + SEM; * *p* < 0.05, *** *p* < 0.001.

The OIM were significantly lighter than the WT ones during the entire experiment (*p* < 0.0001), as highlighted by their weight at 5, 11, and 17 weeks (Figure 1A). There was no gender-related impact on body weight. OIM had an average weekly weight gain of 3.92 ± 2.75% versus 4.63 ± 3.73% for WT ones (*p* = 0.4). Coherently, the femur and the tibia were significantly shorter in the OIM than in WT mice, whereas no significant difference was observed in the length of the humerus between both groups (Figure 1B–D).

### 2.2. Fractures

**Figure 2:**
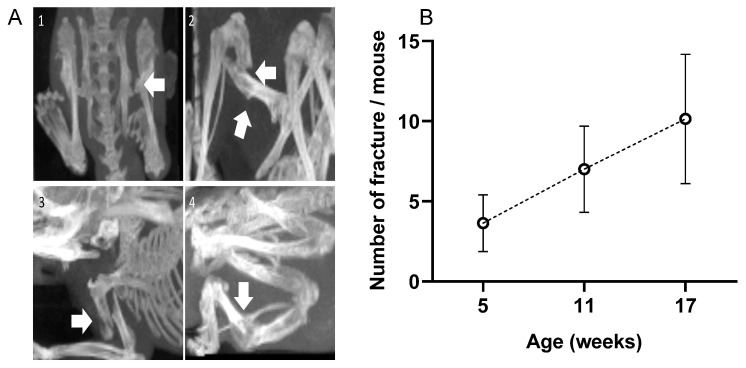
(**A**) 3D scans of OIM showing fractures (arrows); (**1**): femoral neck fracture; (**2**): femoral diaphysis fracture; (**3**): humeral diaphysis fracture; (**4**): tibial diaphysis fracture. (**B**) Total number of fractures per mouse at 5, 11, and 17 weeks of age. Fracture number included the whole body of the 14 OIM, except the vertebrae and the tail. Results are shown with mean ± SD.

Numerous fractures were observed in the OIM with a 3D scan. There were no fractures in the WT group. As visible in Figure 2A, fractures did not only concern the diaphysis but also the femoral neck and deltoid tuberosity. The calcaneus often suffered from alvusion fractures, as did the olecranon of the ulna. As visible in Figure 2B, the OIM had a very high number of fractures as early as 5 weeks of age, and the cumulative rate was consistent over time. Indeed, we observed 3.4 ± 1.6 additional fractures per mouse between 5 and 11 weeks and 3.1 ± 2.2 between 11 and 17 weeks. When considering the main long bones at 17 weeks, 79% of OIM showed at least one humeral fracture, 71% showed one femoral fracture, and only 21% showed a tibial fracture. Other fractures were observed on the pelvis, calcaneum, fibula, and ulna.

### 2.3. pQCT Data

As displayed in Table 1, the bone mineral density of the humerus was significantly lower in OIM than in WT mice (−14.1%). This difference was not observed in the femur and the tibia; however, their cross-sectional area was significantly lower in OIM than in WT mice (−18.86%). The cortical bone area of the three bones was significantly lower in the OIM group, but the cortical area/cross section area (CSA) ratio was significantly lower only in the OIM humerus. Finally, the stress–strain index (SSI) of the OIM femur and tibia was significantly lower than that of WT ones (respectively −40% and −33.9%).

### 2.4. Histology

**Figure 3:**
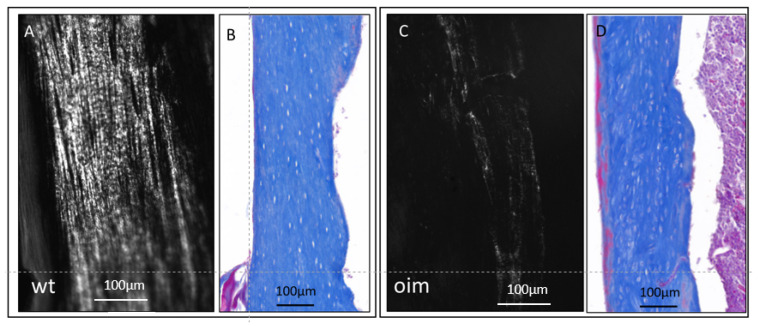
Longitudinal sections through the femur diaphysis of a WT mouse (**A**,**B**) and an OIM (**C**,**D**). (**A**,**C**): unstained sections observed under polarized light, highlighting the collagen organization according to its birefringence. (**B**,**D**): sections stained with Masson’s trichrome and observed in ordinary light microscopy in order to analyze the arrangement of osteocyte lacunae.

In longitudinal sections observed under polarized and ordinary light (Figure 3), the femur diaphysis presented a disturbance in the microarchitecture of cortical bone of OIM, as compared with WT. Collagen appeared disorganized in OIM bone, as attested by the drastic disappearance of the lamellar aspect observed in WT bone (Figure 3A,C). It was associated with modifications in the arrangement of OL, which appeared randomly distributed, more numerous, and somewhat larger in OIM than the WT femur (Figure 3B,D).

**Figure 4:**
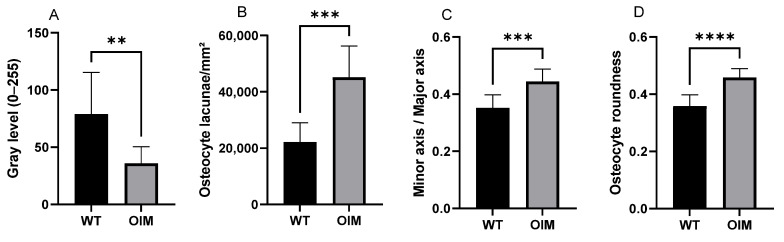
Quantitative analysis of longitudinal sections through the femur diaphysis of WT and OIM. (**A**) Birefringence assessed by the gray value (grayscale 0–255) (polarized light) (WT: n = 13; OIM n = 9). (**B**–**D**) Quantitative parameters of osteocyte lacunae (Masson’s trichrome staining) (WT: n = 10; OIM n = 7): ratio density (/mm^2^) (**B**), minor axis/major axis (**C**), roundness (**D**). Data are shown with mean + SEM; n = 10 in WT group and n = 7 in OIM group; ** *p* < 0.01; *** *p* < 0.001; **** *p* < 0.0001.

Figure 4A displays a significantly lower birefringence of collagen in OIM than in WT femurs in longitudinal sections, as well as in cross-sections. Indeed, the femur sections of OIM showed, respectively, 49.06% (*p* < 0.0001) and 54.59% (*p* < 0.01) lower gray value than WT ones.

Quantitative analyses of longitudinal sections through the femur stained with Masson’s trichrome showed modifications of OL parameters in OIM bones (Figure 4B–D). Osteocyte lacuna density was significantly higher (+103.76%) in the OIM group than the WT one (*p* < 0.001). The elliptic rapport, i.e., minor axis/major axis ratio, was closer to 1 in OIM than WT (*p* < 0.001), and the osteocyte lacuna roundness showed the same trend with a higher roundness in OIM (*p* < 0.0001).

### 2.5. Nano-CT

**Figure 5:**
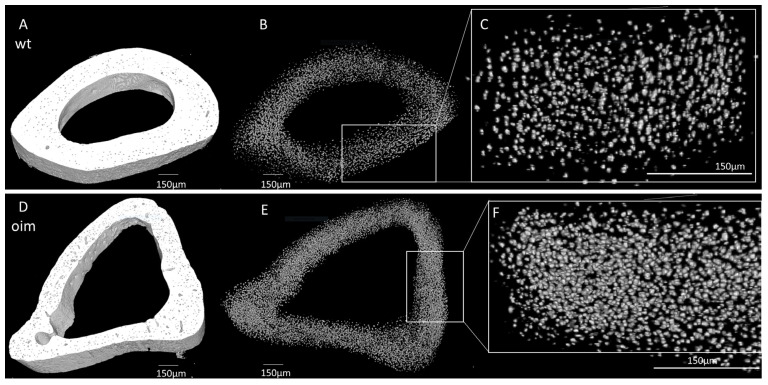
Nano-CT analysis of humerus diaphysis of WT and OIM. (**A**,**D**) Binarized reconstruction of humerus midshaft. (**B**,**E**) 3D reconstruction of the discriminated binarized osteocyte lacunae throughout the midshaft segment. (**C**,**F**) Magnification of 3D reconstruction of the osteocyte lacunae.

As visible in Figure 5A,D, the midshaft contour of the OIM humerus was less regular and less uniform than that of the WT one. There was also an accentuation of the bone margins in OIM. The outer diameter appeared similar in both groups, but cortical thickness was lower, and the medullary cavity was larger in OIM than WT humerus. Selective 3D reconstruction showed more numerous OL in the OIM than in the WT humerus (Figure 5B,C,E,F).

**Figure 6:**
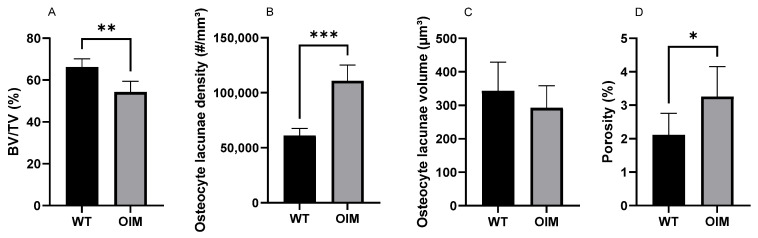
Quantitative nano-CT analysis of WT (n = 7) and OIM (n = 7) humerus midshaft. (**A**) Bone volume/total volume (BV/TV, %). (**B**) Osteocyte lacuna density (#/mm^3^); # = number of lacunae. (**C**) Osteocyte lacuna volume (µm^3^). (**D**) Porosity of cortical bone due to osteocyte lacunae (%). Data are shown with mean + SEM; * *p* < 0.05; ** *p* < 0.01; *** *p* < 0.001; n = 7 in both groups.

The nano-CT quantitative analysis highlighted a significantly lower bone volume/total volume ratio (BV/TV) in the OIM group (−11.74%) than the WT one (*p* < 0.01) (Figure 6A). The quantification of OL parameters confirmed that the OIM humerus had a significantly higher osteocyte lacuna density (+80.01%) than the WT one (*p* < 0.001) (Figure 6B). The volume of the OL was not different between groups, but the porosity due to osteocyte lacunae was significantly higher in OIM bone (+1.14%) (Figure 6C,D).

## 3. Discussion

This study highlighted the combined disorganization of collagen fibers and OL in the long bones of OIM, a murine model of type III OI. Furthermore, the usual bone mass parameters showed different variations between the humerus, femur, and tibia, as was the case of the fracture number.

The general OI features of the OIM were attested by clinometric data, fracture assessment, and long bone densitometry (pQCT).

As observed in previous studies, OIM were consistently lighter than their WT counterparts throughout the experiment [18], which indirectly assessed their small size. It is worth noting that both OIM and WT mice exhibited higher body weight than those reported by Shi et al. [19] and Cardinal et al. [6]. Although they were lighter than WT mice, OIM increased their weight in the same proportions as WT from weeks 5 to 17. In the absence of the difference between the mouse strains, this improvement could be associated with a low number of in vivo manipulations as well as particular attention given to animal housing and care (Refinement). Despite the frail phenotype, there was low mortality in our study since only one mouse died during the experiment.

Like Cardinal et al. [6] and Bargman et al. [20], we measured significantly shorter tibia and femur in OIM than WT mice at 17 weeks. However, we did not observe the same smaller size of the humerus as Cardinal et al. [6], which could be partly explained by the difference in the measurement method (caliper versus X-rays). It can be specified here that an increase in the arm-span/standing height ratio was reported in patients with severe osteogenesis imperfecta [21]. However, in the absence of data about the mouse length due to scoliosis and deformities, it is impossible to extrapolate these differences in long bone lengths to the general size of the mice.

The number of fractures included the humerus, femur, and tibia, as well as the fractures or deformation of the pelvis, calcaneum, ulna, and fibula. Vertebral fractures, not easily distinguishable, were not included. The use of 3D scans instead of 2D X-ray images made it possible to observe multiple fractures on the same bone, such as fractures of the femoral neck and diaphysis or the deltoid tuberosity and humeral diaphysis. These fractures were not always healed between consecutive scans. Our 3D methodology can also explain the higher numbers obtained, as compared with the data of other authors at 4 to 6 weeks of life, i.e., 1.2 to 1.8 fractures per mouse [6,22] and at the end of the experiment.

Densitometric pQCT data reflected the fragility of these OIM long bones in different ways. Indeed, BMD was significantly lower only in the OIM humerus than WT one, whereas cross-sectional area (CSA) and cortical bone area were significantly lower in the OIM femur and tibia than WT ones. These data do not correspond to the low BMD previously obtained in the femur and the tibia of OIM [6] and could reflect a certain heterogeneity between these long bones, as well as an effect of age since the mice were 3 weeks older in the present study. Furthermore, BMD was measured only in the midshaft, and potential variations in the rest of the diaphysis cannot be excluded.

The lower CSA and cortical bone area of OIM femur and tibia led to a significantly lower Stress–Strain Index (SSI) than that of WT bones, which indirectly assessed their fragility, as it was illustrated with 3-point bending test in previous studies [6,22]. The significantly lower cortical bone area of OIM humerus without variation of CSA is evocative of a lower cortical thickness and larger medullary cavity, which was evidenced in micro-CT analysis. This observation, added to the reduction in BV/TV, is consistent with the high number of humerus fractures, in spite of the absence of variation in SSI, calculated from BMD and diaphysis radius squared.

These variations in morpho-densitometric data of the cortical bone among the long bones of OIM draw attention to the need to evaluate different skeletal sites, including trabecular ones, in order to further characterize pathological and therapeutic changes. They also suggest the contribution of other non-quantitative factors in bone fragility, factors more directly linked to collagen abnormality and the disruption of osteoblastic lineage cells.

The lower birefringence of OIM collagen fibers observed with polarized light microscopy attested to a loss of lamellar bone organization compared to WT cortical bone. In equine metatarsal bone, mechanical properties were shown to vary according to the orientation of lamellar bone, assessed by collagen fiber birefringence [23]. The lower collagen birefringence in the OIM femur suggests a woven bone-type organization, such as has been described in the bones of patients with lethal perinatal type II and progressively deforming type III OI, with a mosaic lamellar/woven pattern in the latter [24]. In longitudinal sections through two-thirds of the femoral diaphysis, as well as in cross-sections, the cortical bone of the OIM was mainly constituted of this poorly birefringent bone tissue. This immature type of tissue is made of abnormal collagen that lacks enzymatic cross-links, which leads to a loss in toughness and an increased risk of fracture [25]. The poor lamellar organization of collagen fibers also impacts bone mineralization, as woven bone is known to be possibly hypermineralized [24]. The high mineralization of cortical bone from 8 weeks in OIM was previously found to be associated with low stiffness [26].

As studied in equine, ovine, and murine bone, the arrangement of the osteocyte network is closely interdependent with the organization of the extracellular matrix [27]. Consistent with the low birefringence of cortical bone, longitudinal sections observed under ordinary light showed a poorly oriented distribution of OL throughout the cortical bone of the OIM femur, as is the case in the woven bone of OI patients [28].

This qualitative observation was further confirmed by the significantly higher density of OL in the OIM femur and humerus than in WT ones, quantified in 2D histology and 3D NanoCT, respectively. Similarly, a higher relative surface of osteocytes was reported in the bones of OI patients [24,28,29] and in healing bones [29] in correlation with the woven bone type of the tissue. To our knowledge, only Carriero et al. [30] studied OL 3D organization in the OIM humerus and tibia with Synchrotron CT. They focused their observations on the posterior tibial and the lateral humeral mid-diaphysis from three WT and three OIM and pooled the data of both bones in each group. We obtained similar significant differences in the density of OL between OIM and WT in both 2D histological (femur) and 3D nano-CT (humerus) analyses. These data highly suggest a similar disorganization of collagen matrix in humerus cortical bone, although its birefringence was not analyzed here. Logically, the higher OL density significantly increased the specific porosity related to the osteocyte lacunae. However, this difference of 1.14% only partly contributed to the reduced BV/TV (−11.74%) observed in the humerus cortical bone. Increased number and or size of vascular canals could also contribute to the reduced BV/TV of OIM cortical bone, as highlighted by Carriero et al. [30].

Although we could not use Nano-CT data to analyze 3D lacunar shape because of the resolution (voxel size 1.667 µm), OL analyzed in the histological sections of femurs showed a significantly more pronounced elliptic shape and a higher roundness ratio in OIM than WT mice, in agreement with the more spherical OL observed by Carriero et al. [30] in OIM bone. Regardless of OI collagen mutation, age also seems to have an impact on characteristics of OL which tended to be less numerous and more spherical in younger patients or mice than old ones [31].

Since the arrangement of type I collagen and the alignment of the osteocyte network in the bone matrix are closely interdependent, any mutation of the genes encoding collagen is expected to disrupt the organization of the osteocyte network, which has to be taken into account when investigating collagenopathy.

## 4. Materials and Methods

### 4.1. Mice

Homozygous OIM/OIMB6C3Fe (strain a/a-Col1a2 OIM/J [17]) and homozygous wildtype mice (strain B6C3Fe-a/a+/+, SN1815) (Charles River Laboratories, 69592 L’Arbresle, France) were used in this experiment. All the conditions for animal housing were respected according to the Belgian Federal Law for Animal Care. The Animal Studies Committee of Université Catholique de Louvain allowed this work with the reference 2021/UCL/MD/047.

### 4.2. Schedule of Procedure

After genotyping, mice were included in the osteogenesis imperfecta group (OIM; seven male and eight female mice) or wildtype one (WT; eight male and nine female mice). Mice were weighed weekly between 5 and 17 weeks of age and scanned with 3D in vivo scan NanoSPECT/CT^PLUS^ (Mediso, Budapest, Hungary) under sevoflurane inhalation anesthesia at 5, 11, and 17 weeks of age. At 17 weeks, mice were euthanized with isoflurane. The right humerus, femur, and tibia were dissected, and bones showing fractures on scans were excluded from the study. The preserved bones were measured with a vernier caliper and fixed in formaldehyde. Because of no significative differences between males and females, the data were pooled in each group without gender distinction.

### 4.3. Fracture Count

Scans performed at 5, 11, and 17 weeks of age allowed to identify and count the fractures. Bone deformation, fracture, and callus formation were considered fractures. A deformation observed after an earlier fracture was not counted as a new one. Since the tail was not systematically radiographed, fractures of the tail were not counted.

### 4.4. Peripheral Quantitative Computed Tomography (pQCT)

The right humerus, femur, and tibia were analyzed ex vivo using a pQCT Research SA+ scanner equipped with the XCT 5.4 built-in software (Stratec Medizintechnik GmbH, Pforzheim, Germany). Three parallel transverse slices through the midshaft were acquired with a distance of 0.03 mm between each other. Voxel size was 0.07 mm × 0.7 mm × 0.7 mm, and the threshold was 570 mg/cm^3^ for cortical bone and 280 mg/cm^3^ for general bone parameters. Bone Mineral Density (BMD), Cross-Sectional Area (CSA), Cortical Bone Area (Cort. area), and Stress–Strain Index (SSI, an indirect measure of bone torsional strength) were blinded and analyzed. For each parameter analyzed, the mean value consisted of the mean value of the three slices of each bone. The bones with fractures were not analyzed.

### 4.5. Histology

Right femurs without fracture were decalcified, embedded in paraffin, and sliced into longitudinal and transverse sections. Three unstained longitudinal (proximal two-thirds of the diaphysis) and transverse slices (distal third of the diaphysis) of each bone were observed under polarized light with Axioplan microscope (Zeiss, Jena, Germany) with 1.25 mm objective lens. After digitalization, collagen birefringence was assessed with ImageJ/Fiji software (v1.54f). The images were 8-bit transformed, and the birefringence of cortical bone was measured as the mean of the grey levels between 0 (black) and 255 (white). Two longitudinal slices of each bone were stained with Masson’s trichrome, observed under ordinary light microscopy, and scanned with an SCN400 slide scanner (Leica Biosystems, Wetzlar, Germany) at 20× magnification. OL were visually differentiated from vascular lacunae by their specific staining and were manually counted in cortical bone. The scans were 8-bit transformed, binarized via Fiji/BoneJ (v1.54f), and OL were hand-selected. Lacunae were analyzed with the ‘elliptic fit’ function. The ratio of the minor axis of the ellipse to the major axis was calculated. A value of 1 indicates that the two axes have the same length and, therefore, that the osteocyte lacuna is close to a circle. The roundness function was also used, which is the ratio of the perimeter of a perfect circle to the object’s actual perimeter. It represents how close the object is to the shape of a perfect circle. A roundness value close to 1 indicates a more circular shape.

### 4.6. Nano-CT

Humeri without fracture were scanned under Nano-CT scan (Phoenix NanoTom S, GE Measurement and Control Solutions, Wunstorf, Germany), and 3000 images were taken in mid-diaphysis with a voxel size of 1.5 × 1.5 × 1.5 µm. The source, equipped with a tungsten target, was operated at 85 kV and 70 μA with an exposure time of 750 ms. An aluminum filter of 0.2 mm was applied to reduce beam hardening. All microCT datasets were reconstructed with the Datos|x software version 2.7.0 (GE Measurement and Control Solutions, Wunstorf, Germany) and exported as XY slices (.tiff). After reconstruction, all datasets were normalized to one sample to harmonize all gray levels. The process of normalization was conducted using a Matlab Script 7.0 (The MathWorks, Inc., Natick, MA, USA) [32,33]. A total of 150 slices at the distal part of the deltoid tuberosity were stacked via Fiji/BoneJ (v1.54f). OL were analyzed with Fiji/BoneJ as described by Carriero et al. [30]. The stack was binarized. First, the bone volume/total volume was measured via Fiji/BoneJ routine ‘analyze particles’. For osteocyte lacunae, Fiji routine ‘analyze particles’ was processed to find and delimit all the holes in the binarized stack and reconstruct the particles. Particles were considered OL with a threshold between 50 and 1500 µm^3^ as defined by Carriero et al. [30]. Then, the holes were filled with the option ‘fit holes’, and OL were identified. BoneJ ‘particle analyzer’ routine was processed and allowed to obtain all parameters needed, including lacuna volume and lacuna density.

### 4.7. Statistics

Statistics were performed with GraphPad6.0 using a *t*-test or corresponding non-parametric tests such as the Mann–Whitney test and Student’s *t*-test. Normality was verified with the Shapiro–Wilk normality test, Kolgomorov–Smirnov normality test, and QQ Plot. Results are expressed as mean ± SD or SEM. Data were considered significant if *p* < 0.05.

## 5. Conclusions

This study highlighted qualitative and quantitative alteration of bone tissue in OIM long bones, such as reduced collagen birefringence and disorganized osteocyte lacunae, which could contribute to bone fragility in addition to low BMD and/or impaired geometric data. These observations draw attention to the osteoblastic/osteocytic cell-matrix interactions that influence each other. Further studies would consider these parameters in correlation with mechanical testing, bone matrix composition, osteocyte–dendritic network, and osteocyte viability and activity.

## Figures and Tables

**Table 1:** Peripheral quantitative computerized tomography (pQCT) parameters of humerus, femur, and tibia midshaft of OIM and WT mice.

	Humerus		Femur		Tibia	
	WT (n = 15)	OIM (n = 8)	WT (n = 14)	OIM (n = 10)	WT (n = 15)	OIM (n = 13)
BMD (mg/cm^3^)	813.8 ± 27.8	699.1 ± 54.6 ****	785.6 ± 67.0	730.7 ± 59.9	868.0 ± 48.3	881.2 ± 59.4
CSA (mm^2^)	1.19 ± 0.10	1.14 ± 0.20	2.05 ± 0.30	1.64 ± 0.10 **	1.36 ± 0.30	0.94 ± 0.20 ***
Cort. area (mm^2^)	0.76 ± 0.10	0.65 ± 0.10 *	1.18 ± 0.10	0.91 ± 0.10 ****	0.88 ± 0.10	0.64 ± 0.10 ****
Cort. area/CSA (%)	64.69 ± 2.40	57.61 ± 4.10 ****	58.36 ± 4.80	55.20 ± 3.60	65.32 ± 5.30	68.59 ± 4.50
SSI (mm^3^)	0.20 ± 0.10	0.19 ± 0.10	0.55 ± 0.10	0.36 ± 0.10 ****	0.31 ± 0.10	0.18 ± 0.10 ***

BMD = Bone Mineral Density; CSA = Cross-Sectional Area; Cort. area = Cortical Bone Area; SSI = Stress Strain Index. Results are expressed as mean ± SD. * *p* < 0.05; ** *p* < 0.01; *** *p* < 0.001; **** *p* < 0.0001.

## Data Availability

Data are contained within the article.
